# Supplementary material for: Accuracy of latest large language models in answering multiple choice questions in dentistry: A comparative study
Source: PLoS One. 2025 Jan 29;20(1):e0317423. doi: 10.1371/journal.pone.0317423 (PMC11778630; doi:10.1371/journal.pone.0317423)
Supplement: Attachment — Submitted filename: Response to reviewer R2.docx [file pone.0317423.s003.docx]

December 12^th^, 2024

Dear Editorial Board, PLOS ONE,

We have revised the manuscript thoroughly according to the comments of the reviewers. Any revisions made in our manuscript document were highlighted in red. Please help us review the manuscript again.

Sincerely,

---------------------------------

Reviewer #1: The authors have addressed all my concerns.
Congrats! Before the final acceptance, please polish the English writing.

Thank you for your commendation. We have carefully reviewed the manuscript for grammar and language errors.

--------------------------------

Reviewer #2: The article aims to evaluate the accuracy of responses provided by current-generation Large Language Models (LLMs) to multiple-choice questions (MCQs) in the field of dentistry. The study is interesting both in terms of its chosen subject and methodological approach. Considering the lack of literature on the potential impact of LLMs in dental education and clinical practice, this work may provide a significant contribution. Methodologically, the performances of different LLMs have been comparatively and statistically analyzed. Overall, the article is well-structured, fluent, and written in a clear language.

I have only two suggestions.

First, the authors emphasize that they always used the most up-to-date LLM. However, given the rapid evolution of LLMs, it should be made clearer that this emphasis applies to August 2024. Currently, it is only mentioned once in the methodology section and not stated in the abstract.

Thank you for your comment. We have added this clarification to the abstract:

This study evaluated six of the latest LLMs as of August 2024, including ChatGPT 4.0 omni (OpenAI), Gemini Advanced 1.5 Pro (Google), Copilot Pro with GPT-4 Turbo (Microsoft), Claude 3.5 Sonnet (Anthropic), Mistral Large 2 (Mistral AI), and Llama 3.1 405b (Meta).

The other suggestion concerns clinical reasoning mentioned within the article. There are studies in which LLMs are not only used to solve questions but also to produce questions utilizing clinical reasoning. At least a brief mention of this point could be included in the introduction regarding its relevance for medical/dental education. There are studies in the literature on LLM prompts designed to measure clinical reasoning skills. A recent study has even generated Script Concordance Test questions, for example:

Kıyak, Y. S., & Emekli, E. (2024). Using Large Language Models to Generate Script Concordance Test in Medical Education: ChatGPT and Claude. Spanish Journal of Medical Education, 6(1). <https://doi.org/10.6018/edumed.636331>

Thank you for your suggestion. We have revised the introduction section and added your suggested reference:

Beyond their use in answering clinical questions, LLMs are also being explored for their potential to generate questions that assess clinical reasoning skills, a crucial aspect of medical and dental education [4].
